# Effectiveness of social and behavioral change communication intervention to promote the use of 7.1% chlorhexidine for umbilical cord care in hard-to-reach rural Bangladesh: A mixed method study

**DOI:** 10.7189/jogh.11.04006

**Published:** 2021-01-31

**Authors:** Lutfe Ara, Md Al Amin, Waseq Billah, Shohel Mahmud, Riyasad Iqbal, Tarannum Rahman, Md Ehsanul Haque Tamal, Eben Kenah

**Affiliations:** 1 Clinical Governance and Systems, icddr,b, Dhaka, Bangladesh; 2 Biostatistics Division, College of Public Health, The Ohio State University, Columbus, Ohio, USA

## Abstract

**Background:**

Developing countries account for 99.0% of the 2.7 million neonatal deaths occurring worldwide each year. Umbilical cord infection contributes greatly to this predicament, but evidence shows that 7.1% chlorhexidine solution (CHX) can substantially reduce the risk of infection. To address this challenge, this study aimed to determine the effect of a social and behavioral change communication (SBCC) intervention on promoting the use of WHO recommended CHX as well as on improving the knowledge, attitude, and practices of rural communities regarding umbilical cord care in hard-to-reach areas of Bangladesh.

**Methods:**

A pretest-posttest quasi-experimental study was conducted in two unions of Jamalpur district during 2017-2019 among 748 pregnant women in their third trimester. The SBCC intervention was implemented through town-hall meetings (n = 3), community meetings (n = 30), and door-to-door meetings (n = 22 223) in Dangdhora union, which served as the intervention group, while Hativanga union was kept as a real-time comparator group. Qualitative data were collected from a total of 200 respondents, where 100 participants were chosen from both intervention and control groups. Statistical analysis was carried out in R and outcomes with *P* values less than 0.05 at 95% confidence intervals (CIs) were presented.

**Results:**

Following SBCC intervention, significant (*P* < 0.001) improvements were observed in the intervention group with regards to the primary objective: CHX use increased from 1.07% to 57.80%, while CHX use decreased from 1.6% to 0.0% in the control group. Meaningful improvements were also observed in relation to knowledge (29.0% to 43.0%), attitude (53.0% to 90.0%), and practices (25.0% to 70.0%) of rural communities regarding cord care. Marked improvements were also observed in the intervention group related to understanding causes of cord infections; importance of cord cleanliness; use of antiseptic and other preventive measures; care-seeking behavior; and ensuring hygienic childbirth.

**Conclusions:**

This pioneer study revealed that SBCC interventions led to an increase in CHX use and improved the knowledge, attitude and practices of Bangladeshi communities regarding cord care and cord infection. This indicates that SBCC intervention is indeed an effective and feasible method for reducing infant mortality rates in hard-to-reach populations and achieving SDG goal 3.2.

Neonatal mortality remains a key challenge facing most low- and middle-income countries (LMICs) and so reducing neonatal mortality rates (NMR) is fast becoming one of their top public health priorities [1]. In 2017, 4.1 million infants died within 1 year and 2.5 million newborn deaths occurred within 30 days of birth [2]. With an average of 7000 newborns dying every day, 1 million neonatal deaths occur within 24 hours after delivery and almost a million die within the first 6 days [2]. One-in-five of these newborns die due to various types of infections, which is the third leading cause of neonatal mortality and is responsible for 48% of all neonatal deaths during the late neonatal period (7-27 days after birth) [3-5]. Almost all of these deaths (99%) occur in LMICs where home deliveries are prevalent. In Sub Saharan Africa, South and Southeast Asia, over 70% of all births are taking place at home [6,7]. Furthermore, home deliveries consisted of 46.5%, 43.8%, 34.3%, and 15.4% of all births in Pakistan, Afghanistan, Nepal and India respectively and 2/3 of all home deliveries in South Asia are assisted by traditional birth attendants (TBAs) [5,8]. This, combined with traditional misinformed practices of applying inappropriate substances (cow dung, mustard oil, garlic, boric powder, turmeric etc.) onto the freshly cut umbilical stump, leads to increased cord infections in rural communities [1,9].

In Bangladesh, the child (under 5) mortality rate has fallen dramatically from 151 per thousand live births in 1990; to 65 per thousand live births in 2007; and to 23 per thousand live births in 2015 [10]. Nevertheless, desired progress was not achieved with regards to neonatal mortality (NMR) as the latest report from Bangladesh Demographic and Health Survey (BDHS) found the average NMR over the past five years to be 30/1000 [11]. Recently, the neonatal and infant mortality rates have decreased to 17.1 and 2.5 per 1000 births respectively in 2018, although the child (under 5) mortality rates rose to 30.2 per 1000 [12-14]. Furthermore, around 73 697 infants and 50 244 neonatal deaths had occurred, constituting 83.1% and 56.6% of total under-5 deaths [14-17].

Almost 50% of pregnant women in Bangladesh opt for home deliveries and over 56% of those deliveries are assisted by family members or untrained TBAs and take place in unhygienic conditions – leading to a greater number of newborn deaths [11,15]. Around 1 in 5 of these deaths are caused by infections (respiratory infections, neonatal umbilical cord infection, and tetanus) [18]. Cord infections are the result of pathogens transferred from the environment, hands of caretakers, non-sterile cutting instruments, etc., and can lead to sepsis, omphalitis and even death [1,3,19,20].

Community-based randomized trials conducted in Bangladesh, Nepal, Pakistan, and some African countries have proven that 7.1% chlorhexidine solution (CHX) is more effective in reducing umbilical cord infections compared to dry cord and other antiseptics [21]. Its application reduces NMR by 15% and incidence of omphalitis by almost 30% in high-mortality settings [22]. Consequently, the WHO included “7.1% chlorhexidine digluconate solution or gel, delivering 4% chlorhexidine” in the “WHO Model List of Essential Medicines for Children” for umbilical cord care [2]. Considering the current NMR, the government of Bangladesh (GoB), through the Ministry of Health and Family Welfare (MoH&FW), has recommended the single application of 7.1% CHX when the umbilical cord is cut or within 24 hours after birth and is currently administering a guideline formulated to implement this mandate [1,23-26]. This brings the National Health Policy in line with Every Newborn Action Plan (set by the WHO and UNICEF) adopted by the GoB [27].

Contemporary evidence from a community-based cluster-randomized trial assessing the impact of cord cleansing with CHX on NMR, shows that chlorhexidine is currently not being used by rural communities in Bangladesh [3]. However, no prior studies were found that provided SBCC interventions to promote the use of CHX among rural communities in Bangladesh. Thus, this pilot study will be the first of its kind in South Asia to assess the rate of CHX use and the improvement in knowledge, attitude and practices (KAP) regarding umbilical cord care among communities living in hard-to-reach areas, both before and after promoting a low-cost, effective, and evidence-based SBCC intervention in hard-to-reach areas.

## METHODS

### Study design and participants

A pre-posttest quasi-experimental mixed-method study was carried out from September 2017 – October 2019 at the two unions (ie, the lowest rural administrative unit in Bangladesh) of Jamalpur District in three phases: (i) pre-intervention, (ii) intervention, and (iii) post-intervention to assess the effectiveness of SBCC intervention to promote KAP and use of CHX for umbilical cord care in rural communities. The target population for this study included pregnant women; their family members; TBAs, local pharmacists, and influential local personalities (teachers, religious leaders, village heads etc.).

Dangdhora and Hativanga unions (lowest administrative unit), with populations of 39 977 and 20 652 respectively, were selected purposively as the study sites [28,29]. Situated approximately 45 km away from the nearest city, these are two of the most remote areas of Jamalpur District. These unions were deemed as being hard-to-reach since their transportation, communication and utility infrastructures were underdeveloped; they contained no secondary or tertiary level hospitals; and had limited to no access to primary health care facilities [30,31]. Participants from Dangdhora served as the intervention group and received SBCC intervention, while participants from Hativanga served as the control group and received no intervention.

### Sample size

A sample of 187 pregnant women in their third trimester was selected from each union at each phase to assess the rate of CHX use. Therefore, a total of 748 respondents were selected from both unions (with 374 being selected from both unions during pre- and post-intervention phases), wherein the required sample sizes for each phase in each union were approximated as:


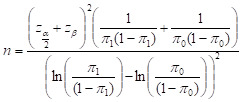


to detect the difference between probabilities π_0_ and π_1_ with power β at significance level α. To account for 10% non-participation, this sample is divided by 0.90. We chose this sample size to detect a difference of 15% point between pre- and post-intervention cumulative incidence of umbilical cord infections with 90% power at a significance level of 0.05.

To assess the KAP of respondents on umbilical cord care, data was collected from a total of 200 respondents. These included 15 postnatal mothers (n = 15); 15 grandmothers or mothers-in-laws (n = 15); 5 influential persons (n = 5), 10 TBAs (n = 10) and 5 local medicine shopkeepers (n = 5). Thus, 50 respondents were purposively selected from each union for the pre- and the post-intervention phases respectively.

### Intervention procedure

#### Pre-intervention phase (4-month):

Pregnant women were enrolled in the study by the Field Research Assistants (FRAs) after obtaining written informed consent. All data related to delivery and umbilical cord care were collected and entered in a case report form (CRF) within 24 hours after birth; and also, on the 7th, 15th, and 28th day from birth. Omphalitis and neonatal deaths were also recorded in the CRF, and the Project Research Physician (PRP) cross-checked all data sets.

#### Intervention phase (4-month):

Social and Behavioral Change Communication (SBCC) model was deployed by organizing 3 town hall meetings; 30 community meetings; and 22 223 door-to-door visits at Dangdhora union. During these meetings, participants were made aware about the severity of cord infections and how 7.1% CHX can prevent infections and deaths in neonates. Meanwhile, the TBAs and community-based skilled birth attendants (CSBAs) were advised to apply this solution. Participants from Dhangdhora Union received this intervention while those from Hativangha Union were kept as a real time comparator group and were not involved in any such activities.

#### Post-intervention phase (4-month):

The post-intervention phase was carried out in a similar fashion to the pre-intervention phase in both Unions.

### Data collection, definition of follow-up times, events, and treatments

Data were collected by FRAs using closed-ended questionnaires and recorded in CRFs through one-on-one interviews while, PRPs inspected newborns for cord infections. The follow-up time for the study to observe the use of 7.1% Chlorhexidine began once the umbilical cord was cut and continued until 28 days after birth to identify symptoms of omphalitis. Babies were assumed to be at risk of developing omphalitis from birth until the first of the following: onset of omphalitis; falling of the umbilical cord; or the end of follow-up.

#### Data analysis

The pre- and post-intervention data from both intervention and control groups were analyzed in a comparative model. Statistical analysis was carried out in R. To test the null hypothesis of no difference in differences, we used three variables: *intervention* equals one in the intervention village (Dangdhora) and zero in the control village (Hativanga), *endline* equals one at endline and zero at baseline, and *interaction* equals the product of intervention and endline. The difference between baseline and endline in the control village is:

(*β_0_* + *β_endline_*) −  *β_0_*,

and the difference between baseline and endline in the intervention village is

(*β_0_* + *β_endline_* + *β_intervention_* + *β_interaction_*) − (*β_0_* + *β_endline_*).

Thus, the difference-in-differences is:

(*β_endline_* + *β_interaction_*) −  *β_endline_* = *β_interaction_*

This approach works for all of the regression models used in our analyses, and these models can be adjusted for additional covariates as needed.

To estimate the difference-in-differences in the use of 7.1% chlorhexidine, we used a linear regression model in which the outcome was an indicator of 7.1% chlorhexidine use and the predictors were intervention, endline, and interaction as explained above. We also fit a model adjusted for mother’s age, place of delivery, and birth assistant (TBA or other).

To estimate the difference-in-differences in the risk of omphalitis, we used survival analysis. For each baby, the variable *time* contained the time between the beginning and end of follow-up or onset of omphalitis, and an event indicator *omph* equaled one if there was an omphalitis onset and zero otherwise. These models included the intervention, endline, and interaction covariates as explained above, and we also adjusted for mother’s age, place of delivery, and birth assistant. We fit a Cox proportional hazards model, but the scaled Schoenfeld residuals indicated violation of proportional hazards. We then fit an accelerated failure time (AFT) model with a log-logistic failure time distribution. The Cox-Snell residuals from this model indicated good fit. When fitting an adjusted model, we chose the function form for mother’s age (linear, quadratic, or cubic) based on Akaike Information Criterion (AIC). We report results as log-logistic rate ratios because these have an interpretation similar to incidence rate ratios.

To estimate the effect of cord care practices and other covariates on the risk of omphalitis, we fit a log-logistic AFT model with a variable for cord care (dry cord or other substances with reference group 7.1% chlorhexidine), clamping instrument (sterile or unsterile), cutting instrument (sterile or nonsterile), neonate sex, mother’s age, place of delivery, and birth assistant. The model was stratified by village, which gave the lowest value of the Akaike Information Criterion (AIC). Cox-Snell residuals indicated good fit for this model as well.

#### Monitoring and evaluation

An independent contract research organization (CRO) was appointed to monitor, assess and evaluate study activities. In total, they conducted 5 visits during the course of the study. At both the pre- and post-intervention phases, they checked the consent forms of the respondents; verified the investigator site review file and validated the information collected in the CRFs. Additionally, they reviewed the activities of the principal investigator and conducted random site visits. Upon completion of the study, a “close out” visit was conducted where the CRO authenticated all data collected. They then locked and sealed the hard copies of the data and place them at the custody of the principal investigator.

## RESULTS

### Quantitative findings

**Figure 1:**
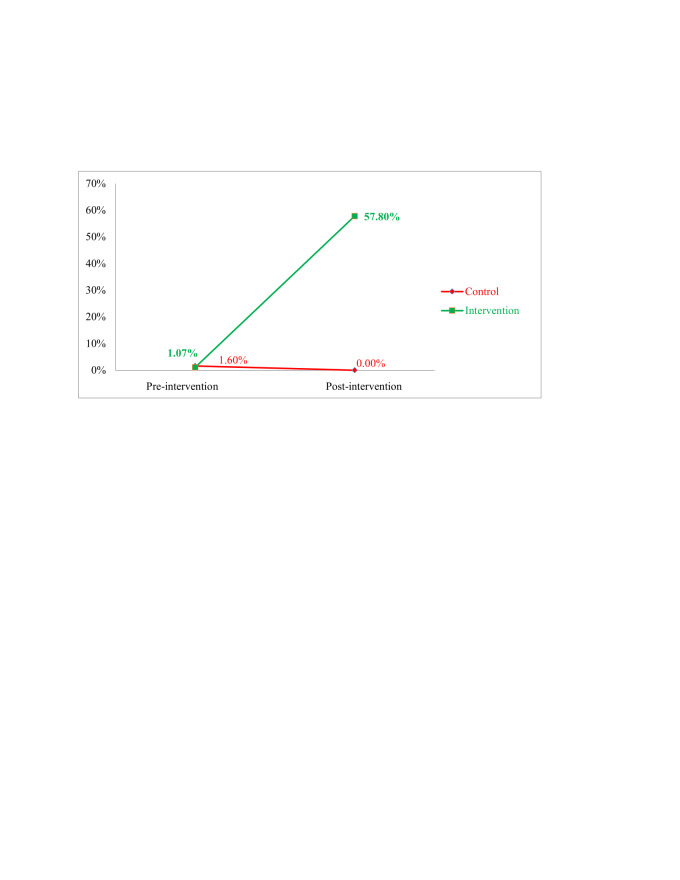
Use of 7.1% chlorhexidine.

Most of the mothers inducted into the study gave birth at home, mostly assisted by the TBAs (**Table 1**). The table also shows that use of health facilities had increased in the intervention group from 9.6% to 11.8%, while in the control group it slightly decreased from 15.5% to 15.0%. The intervention group showed a significant increase in CHX use from 1.07% (95% CI = 0.1% 3.8%) at the pre-intervention period to 57.80% (95% CI = 50.3% 64.9%) at the post-intervention period. Meanwhile, use of CHX had in fact declined from 1.60% (95% CI = 0.3% 4.6%) to 0.0% (95% CI = 0.0% 2.0%) in the control group (**Figure 1**).

**Table 1:** Age and birth history of mothers

Variables	Intervention group			Control group		
**Pre-intervention**	**Post-intervention**	***P* value**	**Pre-intervention**	**Post-intervention**	***P*-value**
**Age of the mother:**						
Mean±SD	24.2 ± 5.1	24.8 ± 5.0		23.4 ± 3.8	25.0 ± 4.5	
**Place of delivery:**						
Home	169/187 (90.4%)	165/187 (88.2%)	0.503	158/187 (84.5%)	159/187 (85.0%)	0.889
Health facility	18/187 (9.6%)	22/187 (11.8%)		29/187 (15.5%)	28/187 (15.0%)	
**Home deliveries assisted by:**						
TBA	139/169 (82.2%)	144/165 (87.3%)	0.454	138/158 (87.3%)	152/159 (95.6%)	0.053
CSBA	26/169 (15.4%)	16/165 (9.7%)		10/158 (6.3%)	2/159 (1.3%)	
Family Member	2/169 (1.2%)	3/165 (1.8%)		10/158 (6.3%)	5/159 (3.1%)	
Others	2/169 (1.2%)	2/165 (1.2%)		0/158 (0.0%)	0/159 (0.0%)	

SD – standard deviation, TBA – traditional birth attendant, CSBA – community-based skilled birth attendant

**Figure 2:**
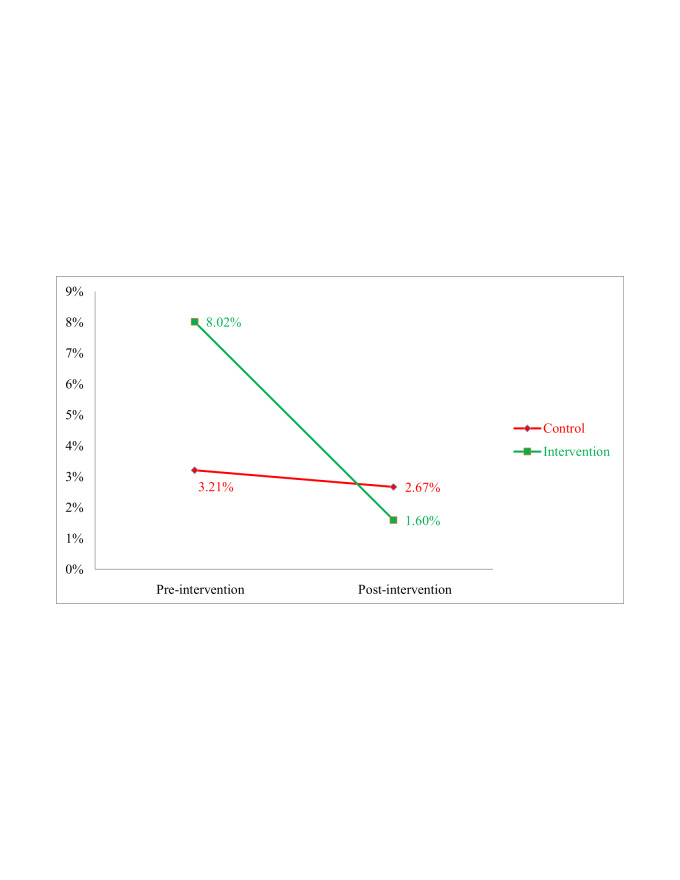
Rate of omphalitis.

The top panel of **Table 2** shows the results of the corresponding linear regression model, which shows that the difference in differences was highly statistically significant after adjustment for the age of the mother (linear), sex of the neonate, place of delivery, and birth assistant. The unadjusted results are very similar. **Figure 2** shows that the rate of omphalitis had substantially decreased from 8.02% to 1.60% in the intervention group. However, no such remarkable change was observed in the control group for this variable. The bottom panel of **Table 2** shows that this difference was highly statistically significant after adjustment for the age of the mother (cubic polynomial), sex of the neonate, place of delivery, and birth assistant. The unadjusted results are very similar.

**Table 2:** Regression coefficients for use of CHX and odds ratios for omphalitis rate parameters

Covariate	Coefficient	Rate ratio	95% confidence interval	*P*-value
**Regression coefficients for the use of CHX (adjusted for age of mother, sex of child, place of birth, and birth attendant)**				
Endline	-0.01		(-0.06, 0.04)	0.684 2
Intervention	-0.01		(-0.07, 0.04)	0.619 3
Interaction	0.58		(0.51, 0.66)	0.000
**Estimated log logistic rate ratios for the omphalitis onset (adjusted for age of mother, sex of child, place of birth, and birth attendant)**				
Endline		0.88	(0.42, 1.84)	0.729
Intervention		1.72	(0.92, 3.20)	0.089
Interaction		0.26	(0.09, 0.80)	0.019

CHX - Chlorhexidine

**Figure 3:**
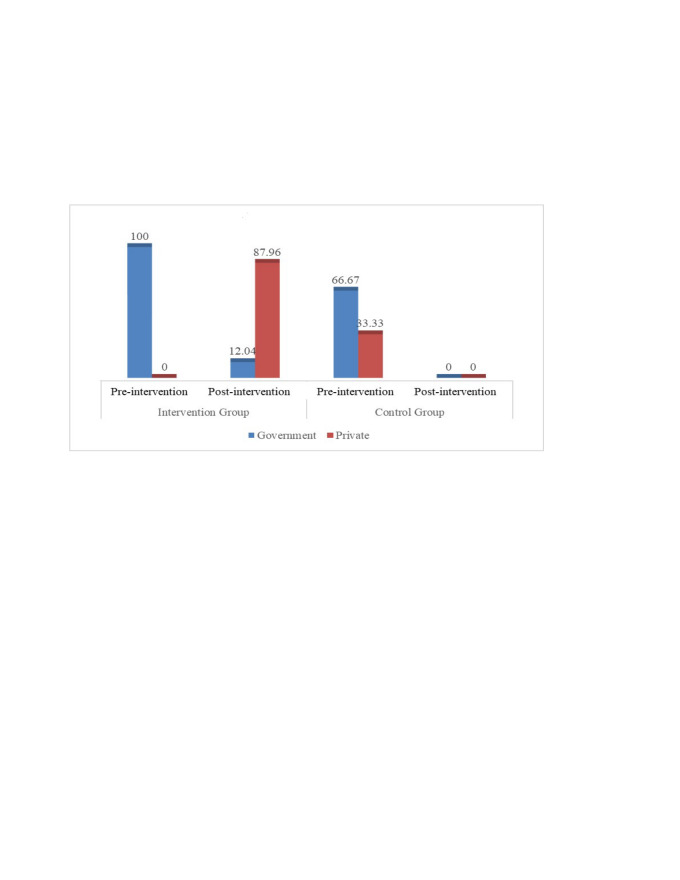
Sources of chlorhexidine.

In the invention group, use of hexisol (10.5% to 15.1%); clips as a clamping instrument (2.7% to 4.3%) and sterile clamping instruments (96.8% to 100%) increased after the intervention (**Table 3**). **Table 4** shows estimated log logistic rate ratios for omphalitis onset by type of cord care, clamping, and cutting adjusted for neonate sex, mother’s age, and village. Dry cord and use of substances other than 7.1% chlorhexidine were significantly associated with higher risk of omphalitis. **Figure 3** shows that government sources were mostly used by participants of both groups to obtain CHX at the pre-intervention phase. However, in the post-intervention phase, participants from the intervention group mostly obtained CHX from private sources.

**Table 3:** Materials and measures taken for cord care

Variables	Intervention group			Control group		
**Pre-intervention**	**Post-intervention**	***P*-value**	**Pre-intervention**	**Post-intervention**	***P*-value**
**Cord cleaning instruments before clamping and cutting**						
Cloth	70/152 (46.1%)	132/185 (71.4%)	0.000	114/161 (70.8%)	128/184 (69.6%)	0.000
Cotton	1/152 (0.7%)	9/185 (4.9%)		3/161 (1.9%)	20/184 (10.9%)	
Hexisol	16/152 (10.5%)	28/185 (15.1%)		18/161 (11.2%)	20/184 (10.9%)	
Savlon	16/152 (10.5%)	0/185 (0%)		8/161 (5.0%)	8/184 (4.4%)	
Water	43/152 (28.3%)	16/185 (8.7%)		15/161 (9.3%)	1/184 (0.5%)	
Other Instruments	6/152 (4.0%)	0/185 (0%)		3/161 (1.9%)	7/184 (3.8%)	
**Clamping instruments:**						
Clip	5/187 (2.7%)	8/185 (4.3%)	0.000	10/187 (5.4%)	2/184 (1.1%)	0.000
Cloth	0/187 (0%)	0/185 (0%)		1/187 (0.5%)	0/184 (0%)	
Thread	182/187 (97.3%)	177/185 (95.7%)		176/187 (94.1%)	182/184 (98.9%)	
**Sterilization of clamping instruments:**						
Sterile	181/187 (96.8%)	185/185 (100.0%)	0.014	148/187 (79.1%)	167/184 (90.8%)	0.001
Non-sterile	6/187 (3.2%)	0/185 (0%)		39/187 (20.9%)	17/184 (9.2%)	
**Cutting instruments:**						
Blade	146/187 (78.1%)	166/185 (89.7%)	0.000	151/187 (80.7%)	140/184 (76.1%)	0.281
Scissor	35/187 (18.7%)	9/185 (4.9%)		20/187 (10.7%)	32/184 (17.4%)	
Surgical Blade	6/187 (3.2%)	10/185 (5.4%)		14/187 (7.5%)	10/184 (5.4%)	
Others	0/187 (0%)	0/185 (0%)		2/187 (1.1%)	2/184 (1.1%)	
**Sterilization of cutting instruments:**						
Sterile	183/187 (97.9%)	185/185 (100.0%)	0.045	165/187 (88.2%)	180/184 (97.8%)	0.000
Non-sterile	4/187 (2.1%)	0/185 (0.0%)		22/187 (11.8%)	4/184 (2.2%)	
**Measures taken for cord care other than 7.1% chlorhexidine:**						
Dry Cord	140/185 (75.7%)	62/79 (78.5%)	0.000	160/184 (87.0%)	159/187 (85.0%)	0.592
Antiseptics	44/185 (23.8%)	10/79 (12.7%)		24/184 (13.0%)	28/187 (15.0%)	
Mustard Oil	1/185 (0.5%)	7/79 (8.9%)		0/184 (0.0%)	0/187 (0.0%)	

**Table 4:** Rate ratios of different covariates for cord care

Covariate	Rate ratio	95% confidence interval	*P*-value
Dry cord	2.80	(1.13, 6.98)	0.027
Non-chlorhexidine substance	3.51	(1.36, 9.05)	0.009
Unsterile clamping	1.69	(0.70, 4.08)	0.242
Unsterile cutting	1.12	(0.34, 3.68)	0.853

### Qualitative findings

#### Knowledge on newborn cord care

Knowledge about leaving the umbilical cord uncovered increased from 28.6% to 42.9% in the intervention group, whereas in the control group it decreased slightly from 37.1% to 34.3%. Moreover, knowledge about applying substances after cleaning the stump also increased from 37.1% to 68.6% in the intervention group. All (100.0%) respondents in the intervention group were aware of cord infection symptoms after intervention, a significant increase from 22.9% during pre-intervention (**Table 5**).

**Table 5:** Respondent’s knowledge on newborn cord care

Variables	Intervention group		Control group	
**Pre-intervention, n (%)**	**Post-intervention, n (%)**	**Pre-intervention, n (%)**	**Post-intervention, n (%)**
**Should the umbilical stump of your baby be covered with a cloth/bandage or uncovered?**				
Covered	24/35 (68.6%)	20/35 (57.1%)	21/35 (60.0%)	23/35 (65.7%)
Uncovered	10/35 (28.6%)	15/35 (42.9%)	13/35 (37.1%)	12/35 (34.3%)
Don’t know	1/35 (2.9%)	0/35 (0%)	1/35 (2.9%)	0/35 (0%)
**After cleaning your baby’s soiled umbilical stump, should any substances be applied to it?**				
Yes	13/35 (37.1%)	24/35 (68.6%)	16/35 (45.7%)	31/35 (88.6%)
No	22/35 (62.9%)	11/35 (31.4%)	19/35 (54.3%)	4/35 (11.4%)
**Do you know the symptoms of cord infections are?**				
Navel swelling and redness	18/35 (51.4%)	19/35 (54.3%)	13/35 (37.1%)	28/35 (80.0%)
Pus discharge and bleeding	9/35 (25.7%)	16/35 (45.7%)	9/35 (25.7%)	7/35 (20.0%)
Don't know	8/35 (22.9%)	0/35 (0%)	13/35 (37.1%)	0/35 (0%)
**Do you know the preventive measures against cord infections?**				
Should use antiseptic or Savlon	1/35 (2.9%)	3/35 (8.6%)	2/35 (5.7%)	9/35 (25.7%)
Maintain cleanliness, Cover navel and proper care	16/35 (45.7%)	10/35 (28.6%)	17/35 (48.6%)	9/35 (25.7%)
Don’t know	17/35 (48.6%)	22/35 (62.9%)	14/35 (40.0%)	17/35 (48.6%)
Avoid sweets	1/35 (2.9%)	0/35 (0%)	2/35 (5.7%)	0/35 (0%)

In this regard, a post-natal mother quoted that:

“I didn’t know that 7.1% Chlorhexidine can save my child from infection. I used to only apply mustard oil on the umbilical cord after cutting it.”

Another post-natal mother (**Box 1**) stated that:

Box 1 **Case study 1**

Mrs. Foolbanu (Pseudonym) is 28-year-old resident of the intervention Union and a mother of 3 children. She did not know that the minimum number of ante-natal visits (ANV) is 4. During the intervention period, she learned about the importance of these visits and realized that anaemia was the reason for her prior miscarriages.

She also previously lacked knowledge regarding symptoms of cord infections and had even applied “cow dung” to the freshly cut umbilical cord stump of her previous 1-month old baby boy. This only aggravated his condition and led to his untimely death. However, recently TBAs had applied chlorhexidine immediately after cutting the umbilical cord of her new-born baby and tied it securely with sterilized materials. She was extremely satisfied with the intervention and believed it to have saved both their lives.

“I didn’t know that my elder son had cord infection which ultimately cost his life. After learning about the symptoms, I have become careful and have used 7.1% Chlorhexidine for my newborn.”

#### Attitudes on newborn cord care

Major improvements were observed in the attitude of local communities regarding cord infections as the proportion of respondents strongly agreeing to it being a serious cause of illness increased from 72.0% to 90.0% and from 86% to 90% in intervention and control group respectively. Participants also showed increased interest (rising from 82.0% to 98.0% in the intervention and from 96% to 98% in the control group) in applying 7.1% Chlorhexidine after birth of newborns. Moreover, the proportion of respondents strongly disagreeing about use of old razor blade rose from 36.0% to 96.0% in the intervention group while, in the control group, this actually decreased from 96.0% to 38.0% (**Table 6**). Accordingly, one of the TBAs stated:

**Table 6:** Respondent’s attitude on newborn cord care

Variables	Intervention group		Control group	
**Pre-intervention, n (%)**	**Post-intervention, n (%)**	**Pre-intervention, n (%)**	**Post-intervention, n (%)**
**A previously used razor blade can be washed and used to cut the cord:**				
Agree	4/50 (8.0%)	0/50 (0%)	0/50 (0%)	6/50 (12.0%)
Disagree	28/50 (56.0%)	2/50 (4.0%)	2/50 (4.0%)	25/50 (50.0%)
Strongly disagree	18/50 (36.0%)	48/50 (96.0%)	48/50 (96.0%)	19/50 (38.0%)
**A dirty umbilical cord can cause infection in your baby:**				
Strongly agree	26/50 (52.0%)	42/50 (84.0%)	27/50 (54.0%)	37/50 (74.0%)
Agree	19/50 (38.0%)	7/50 (14.0%)	3/50 (6.0%)	13/50 (26.0%)
Disagree	0/50 (0%)	1/50 (2.0%)	1/50 (2.0%)	0/50 (0%)
Strongly disagree	5/50 (10.0%)	0/50 (0%)	19/50 (38.0%)	0/50 (0%)
**Umbilical cord infection is a serious cause of illness:**				
Strongly agree	36/50 (72.0%)	45/50 (90.0%)	43/50 (86.0%)	45/50 (90.0%)
Agree	14/50 (28.0%)	5/50 (10.0%)	7/50 (14.0%)	5/50 (10.0%)
**Do people in the community ever use antiseptic or liquid solution or 7.1% chlorhexidine to clean the cord?**				
Yes	41/50 (82.0%)	49/50 (98.0%)	48/50 (96.0%)	49/50 (98.0%)
No	9/50 (18.0%)	1/50 (2.0%)	2/50 (4.0%)	1/50 (2.0%)

“I didn’t know that a used razor blade cannot be used repeatedly for cord-cutting or that it needs to be sterilized completely. I used to clean it with soap and water but now, I boil it and also apply Savlon before use.”

#### Practices on newborn cord care

During pre-intervention, 60.0% and 55.9% of respondents in the intervention and control group respectively, used sterile materials whereas, this number increased to 100.0% for both groups post-intervention. The study found that 70.0% of respondents in the intervention group had applied 7.1% Chlorhexidine to the cord stump after SBCC (increasing from only 25.0% during pre-intervention). Furthermore, TBAs were applying this substance in all cases at the intervention village, whereas use of 7.1% Chlorhexidine actually declined (28.6% to 14.3%) in the control group (**Table 7****).** The interviews yielded similar findings as a TBAs (after receiving SBCC) stated (**Box 2**):

**Table 7:** Respondent’s practices on newborn cord care

Variables	Intervention group		Control group	
**Pre-intervention, n (%)**	**Post-intervention, n (%)**	**Pre-intervention, n (%)**	**Post-intervention, n (%)**
**Were the materials used for tying the cord available at home?**				
Yes	21/35 (60.0%)	30/35 (100.0%)	19/35 (55.9%)	35/35 (100.0%)
No	14/35 (40.0%)	0/35 (0%)	15/35 (43.1%)	0/35 (0%)
**Was something applied on the cord after the cord was cut?**				
Yes	8/35 (22.9%)	25/35 (71.4%)	7/35 (20.0%)	33/35 (94.3%)
No	27/35 (77.1%)	10/35 (28.6%)	28/35 (80.0%)	2/35 (5.7%)
**If “yes”, what was applied on the cord stump?**				
Savlon	3/8 (37.5%)	6/20 (30.0%)	3/35 (42.9%)	24/28 (85.7%)
Hexicord	2/8 (25.0%)	14/20 (70.0%)	2/35 (28.6%)	4/28 (14.3%)
Povidon, Vermilion,Viodin	3/8 (37.5%)	0/20 (0%)	1/35 (14.3%)	0/28 (0%)
Don’t know	0/8 (0%)	0/20 (0%)	1/35 (14.3%)	0/28 (0%)
**If “yes”, who did apply these substances?**				
Mother	2/6 (33.3%)	0/20 (0%)	1/7 (14.3%)	0/28 (0%)
Grandmother	3/6 (50.0%)	0/20 (0%)	2/7 (28.6%)	0/28 (0%)
Traditional birth attendants	1/6 (16.7%)	20/20 (100.0%)	2/7 (28.6%)	28/28 (100.0%)
Others			2/7 (28.6%)	0/28 (0%)
**What do you do if the baby’s cord has redness or swelling or pus formation?**				
Take advice	33/35 (94.3%)	32/34 (94.1%)	33/35 (94.3%)	35/35 (100.0%)
Apply medicine, powder or mustard oil after cleaning with Savlon	2/35 (5.7%)	2/34 (5.9%)	1/35 (2.95%)	0/35 (0%)
Don’t know	0/35 (0%)	0/34 (0%)	1/35 (2.9%)	0/35 (0%)

Box 2 **Case study 2**

Traditional birth attendant (TBA), Mrs. Roxana (Pseudonym) aged 60, has delivered babies at the intervention Union for over 30 years. She had learned everything she knows from her mother who was also a TBA. However, after witnessing some complications during deliveries that led to the death of the mother and/or neonate, she opted to receive training. Previously, she would have only applied Savlon but after attending SBCC interventions, she has started using Hexicord which she now carries to deliveries or orders for it prior to attending the mother. Furthermore, she now ensures that the umbilical cord is cut with a new sterilized blade and is tied by a clean thread.

“Previously, I used povidone but now, I use Hexicord until the cord falls off.’

#### Ante-natal care and birth history of ante-natal mother

A significant increase (80.0%) in attendance of antenatal care (ANC) was observed in pregnant mothers in the intervention group post-intervention. Among them, three-fourth of ante-natal mothers visited ANCs around 1-3 times, whereas the rest (one-fourth) attended 4-6 times (**Table 8**). In this regard, one of the ante-natal mothers said:

**Table 8:** Antenatal care and birth history of mothers

Variables	Intervention group		Control group	
**Pre-intervention, n (%)**	**Post-intervention, n (%)**	**Pre-intervention, n (%)**	**Post-intervention, n (%)**
**Attended ante natal clinics (ANC) during the pregnancy:**				
Yes	11/15 (73.3%)	12/15 (80.0%)	6/15 (40.0%)	11/15 (73.3%)
No	4/15 (26.7%)	3/15 (20.0%)	9/15 (60.0%)	4/15 (26.7%)
**Number of ANC visits attended:**				
1-3 times	9/11 (81.8%)	9/12 (75.0%)	6/6 (100.0%)	7/10 (70.0%)
4-6 times	2/11 (18.2%)	3/12 (25.0%)	0/6 (0%)	3/10 (30.0%)
**Number of months of pregnancy when first attended ANC:**				
1-4 mo	4/11 (36.4%)	4/12 (33.3%)	5/6 (83.3%)	7/11 (63.6%)
5-8 mo	7/11 (63.6%)	8/12 (66.7%)	1/6 (16.7%)	4/11 (36.4%)
**Advice received from ANC clinic:**				
Heavy work prohibited, Take nutritious food, plenty of water	0/11 (0%)	7/12 (58.3%)	0/6 (0%)	8/11 (72.7%)
Take iron tablets and avoid heavy work	6/11 (54.5%)	5/12 (41.7%)	4/6 (66.7%)	3/11 (27.3%)
No advice is given	3/11 (27.3%)	0/12 (0%)	2/6 (33.3%)	0/11 (0%)
Can't remember	2/11 (18.2%)	0/12 (0%)	0/6 (0%)	0/11 (0%)

ANC – antenatal care

“I did not know that the minimum number of antenatal visits should be 4. Had I known, then I would have visited at least 4 times.”

**Table 8** shows that two-thirds of participants reported seeking antenatal care for the first time between 5-6 months into their pregnancy while the rest visited ANCs much earlier (between 1-4 months). Before the intervention, about half of antenatal mothers either did not get any advice (27.3%) or could not remember (18.2%) the advice received from ANCs (**Table 8**). However, this scenario changed for all mothers after intervention.

#### Decision-making and general aspects regarding delivery

Almost all decisions about place of delivery were taken by either the head of the family; or by the breadwinner; or by the male counterpart of ante-natal mothers (**Box 3**). Only one neonatal mother reported taking the decision herself about place of delivery. A grand-mother interviewed stated:

Box 3 **Case study 3**

Md Masud (Pseudonym) is an 80-year-old grandfather of a 1-month old baby boy from the intervention union. He said that his daughter had lost her first baby due to complications during pregnancy. She had survived a seizure but delivered a stillborn baby after not being allowed to go to the Upazila Health Complex (UHC). After attending the intervention, his daughter felt empowered and was able to decide on the place of delivery herself and visited the UHC eight times for ante-natal care (ANC). She was given medication to control her high blood pressure and finally gave birth to a healthy baby at home assisted by a TBA. The TBA applied Hexicord to the freshly cut cord stump and advised to continue application until cord fall.

“I was not given the right to decide the place of delivery. If I was given the choice, then I could have talked to my daughter and her ante-natal period could have gone smoother.”

Trained TBAs from reputed institutions assisted in many deliveries and almost all of them reported that families of ante-natal mothers call them first when labor pains begin. A TBAs stated that:

“We are the first to be contacted in case of home deliveries and so we should be given professional training on safe home deliveries.”

## DISCUSSION

Several studies pointed out that incorporating CHX in cord care practices can significantly reduce cord infections and thereby NMR [1-4]. Therefore, Save the Children (with the support of USAID and MoH&FW) implemented the MaMoni HSS project in 2015 to train CSBAs on using 7.1% CHX. In 2019, a follow up study examined use of CHX in public health care facilities of Bangladesh [23-27]. However, those studies did not implement SBCC interventions nor analyze CHX use in rural households. In contrast, our study assessed the effectiveness of SBCC interventions in promoting use of 7.1% chlorhexidine and improving the knowledge, attitude, and practices of communities relating to cord care in hard-to-reach areas of Bangladesh.

The success of the intervention is evident in **Figure 1**. As there was no reported chlorhexidine use in control villages during the end-line survey, regression models for binary outcomes using log or logit link functions will not converge. Thus, we fit a linear regression model with an interaction term to test the null hypothesis of no difference-in-differences. SBCC intervention increased 7.1% CHX use from 1.07% to 57.8% in the intervention village. While usage declined in the control village, the estimated increase in 7.1% chlorhexidine use in the intervention village is 58.3% compared to what would have occurred without the intervention (**Figure 1** and **Table 2**). This difference is highly statistically significant (*P* < 0.0001) and emphasizes the effectivity of SBCC intervention among rural communities in promoting the 7.1% chlorhexidine usage. The uniqueness of this study in promoting 7.1% CHX through SBCC intervention in rural communities can also be replicated to achieve similar results in other LMICs as well [3,9,20].

The coefficient on *intervention* shows that there was probably a higher risk of omphalitis in the intervention village than there was in the control village (*P* = 0.06) at baseline. The coefficient on *end-line* shows (**Table 2**) that there was a slight decrease in omphalitis risk in the control village, but this was not statistically significant (*P* = 0.76). The coefficient on the interaction term shows that the intervention village had a much larger reduction in omphalitis risk than the control village, with an interaction odds ratio of 0.12 and *P* value of 0.02. These results support the conclusion that the intervention was successful in reducing the risk of omphalitis in the intervention village. Due to the design of the study, it is unlikely that the large apparent effect of the intervention is attributable to confounding (because each village was compared to itself), selection bias, or differential misclassification. This study was the first of its kind to be conducted in South Asia and so these findings will prove beneficial to other developing countries as well.

Our study strongly suggests that chlorhexidine is the safest substance for cord care (*P* = 0.027) which is concurrent with previous studies [3,21,22]. Findings also suggest that using a sterile clamping instrument is relatively more important than using a sterile cutting instrument and that male babies are at a higher risk of developing omphalitis, though these results are not statistically significant (**Table 4**). Nevertheless, use of non-sterile cutting instruments was always discouraged as well. If we fit the same model with two categories for dry cord and other substances, we get very similar results for clamping, cutting, and sex. Additionally, both dry cord care and use of other substances are associated with higher risk of omphalitis (**Table 4**).

The study design does not prevent confounding of the association between reported cord care and risk of omphalitis in the same way that it prevents confounding by groups (**Table 2**). The association between reported chlorhexidine use and omphalitis is strong, so the results of this study support the conclusion that using 7.1% chlorhexidine on the umbilical cord substantially reduces risk of omphalitis. All three analyses showed that SBCC interventions substantially improved use of 7.1% chlorhexidine for cord care and reduced risk of developing omphalitis in neonates.

The study revealed that public sources of CHX (community clinics) were limited compared to private sources (pharmacies, shops etc.). Results show that all respondents in the intervention group during pre-intervention (n = 2, 100.0%) acquired Chlorhexidine from government sources whereas, post-intervention, most (n = 95, 87.96%) respondents acquired it from private sources. Although studies show that CHX supplies at government facilities have rocketed since 2015, our findings reveal rural people are not obtaining CHX from government sources [27]. This indicates that increased awareness about the availability and proximity of private sources (through SBCC) had increased usage of 7.1% chlorhexidine. Thus, the GoB may use SBCC to increase CHX use in rural communities.

Previous studies exhibited knowledge gaps and lack of communication about cord care in rural communities [27,32-35]. However, the number of mothers that demonstrated improved knowledge about keeping the cord stump uncovered increased from 28.6% to 42.9% in the intervention group, whereas the control group displayed a decrease. Furthermore, significant improvement in knowledge on applying substances (37.1% to 68.6%) and on identifying symptoms of omphalitis (77.1% to 100.0%) was recorded in the intervention group post-intervention.

The WHO emphasized the importance of safe cord-cutting to prevent neonatal tetanus or sepsis and ultimately death [33,36,37]. Accordingly, an increase in attitude on cord care in relation to re-using previously used razor blades (36.0% to 96.0%); cleanliness of the umbilical cord (52.0% to 84.0%); and umbilical cord infections (72.0% to 90.0%) was observed in the intervention group. Moreover, in contrast to the control group, attitudes of communities on using antiseptics or 7.1% Chlorhexidine in cord care improved from 82.0% to 98.0% in the intervention group.

Cord care practices had also dramatically improved in all segments after SBCC as the availability of essential materials for cord care in households increased (60.0% to 100.0%) and the practice of applying a substance on the cord stump also rose by almost 50% in the intervention group. These results are congruent with studies conducted in India and Egypt [37-39]. Also, Behavioral Change Communication (BCC) interventions were implemented in 2016 by Nepal to improve knowledge, attitudes and perceptions among health care workers and people living in hard-to-reach areas by qualitatively exploring barriers and facilitators of acquiring chlorhexidine [31]. India took a different approach in 2013 by solely focusing on training government officials to implement SBCC for maternal and neonatal care [40]. Contrary to both, our study was one of the first in South Asia to implement SBCC for promoting CHX use among hard-to-reach rural populations.

The crucial strength of our study is its uniqueness in terms of study aim, place, and intervention methods. No similar study was conducted in context of our country, especially in terms of using SBCC interventions to promote health behavior and practices. However, geographical location of the study groups; diverse socio-economic profiles of respondents; and lack of data about previous pregnancies of respondents are some limitations of this study.

## CONCLUSIONS

This mixed method pilot study shows that SBCC is an effective mechanism for promoting 7.1% chlorhexidine use and for improving KAP of rural communities regarding cord care and cord infection in Bangladesh. The success of SBCC is evident when comparing and contrasting the findings of the intervention and control groups Furthermore, the interventions were able to bring a positive change in the KAP as well as behavior of households in relation to use of chlorhexidine. Nevertheless, further investigations are needed to assess and identify broader and long-term effects of SBCC on larger study groups and in diverse geographical locations. Being a pilot study, the study team recommends that the GoB design and implement a scaled-up and extended action research and intervention program to reap the benefits of SBCC interventions. Finally, the study outcomes are expected to be helpful for policymakers; health care professionals; governmental and non-governmental authorities (domestic and international); and researchers to take effective and efficient actions to promote chlorhexidine. Such initiatives will greatly reduce NMR throughout the world and thereby lead countries to meet SDGs, including Goal 3.2.2.
